# Estimation of the Population Size of Street- and Venue-Based Female Sex Workers and Sexually Exploited Minors in Rwanda in 2022: 3-Source Capture-Recapture

**DOI:** 10.2196/50743

**Published:** 2024-03-15

**Authors:** Elysee Tuyishime, Eric Remera, Catherine Kayitesi, Samuel Malamba, Beata Sangwayire, Ignace Habimana Kabano, Horacio Ruisenor-Escudero, Tom Oluoch, Angela Unna Chukwu

**Affiliations:** 1 African Center of Excellence in Data Science University of Rwanda Kigali Rwanda; 2 Division of Global HIV and TB Global Health Center US Centers for Disease Control and Prevention Kigali Rwanda; 3 Institute of HIV Disease Prevention and Control Rwanda Biomedical Centre Rwanda Ministry of Health Kigali Rwanda; 4 Key Population Surveillance Team, Epidemiology and Surveillance Branch Division of Global HIV/TB, Global Health Center US Centers for Disease Control and Prevention Atlanta, GA United States; 5 Department of Statistics University of Ibadan Ibadan Nigeria

**Keywords:** population size, female sex workers, capture-recapture, 3-source, Rwanda, HIV, surveillance, population, epidemiology, prevention, AIDS, sexually transmitted disease, STD, minor, young adult, sexually exploited minor, children

## Abstract

**Background:**

HIV surveillance among key populations is a priority in all epidemic settings. Female sex workers (FSWs) globally as well as in Rwanda are disproportionately affected by the HIV epidemic; hence, the Rwanda HIV and AIDS National Strategic Plan (2018-2024) has adopted regular surveillance of population size estimation (PSE) of FSWs every 2-3 years.

**Objective:**

We aimed at estimating, for the fourth time, the population size of street- and venue-based FSWs and sexually exploited minors aged ≥15 years in Rwanda.

**Methods:**

In August 2022, the 3-source capture-recapture method was used to estimate the population size of FSWs and sexually exploited minors in Rwanda. The field work took 3 weeks to complete, with each capture occasion lasting for a week. The sample size for each capture was calculated using shinyrecap with inputs drawn from previously conducted estimation exercises. In each capture round, a stratified multistage sampling process was used, with administrative provinces as strata and FSW hotspots as the primary sampling unit. Different unique objects were distributed to FSWs in each capture round; acceptance of the unique object was marked as successful capture. Sampled FSWs for the subsequent capture occasions were asked if they had received the previously distributed unique object in order to determine recaptures. Statistical analysis was performed in R (version 4.0.5), and Bayesian Model Averaging was performed to produce the final PSE with a 95% credibility set (CS).

**Results:**

We sampled 1766, 1848, and 1865 FSWs and sexually exploited minors in each capture round. There were 169 recaptures strictly between captures 1 and 2, 210 recaptures exclusively between captures 2 and 3, and 65 recaptures between captures 1 and 3 only. In all 3 captures, 61 FSWs were captured. The median PSE of street- and venue-based FSWs and sexually exploited minors in Rwanda was 37,647 (95% CS 31,873-43,354), corresponding to 1.1% (95% CI 0.9%-1.3%) of the total adult females in the general population. Relative to the adult females in the general population, the western and northern provinces ranked first and second with a higher concentration of FSWs, respectively. The cities of Kigali and eastern province ranked third and fourth, respectively. The southern province was identified as having a low concentration of FSWs.

**Conclusions:**

We provide, for the first time, both the national and provincial level population size estimate of street- and venue-based FSWs in Rwanda. Compared with the previous 2 rounds of FSW PSEs at the national level, we observed differences in the street- and venue-based FSW population size in Rwanda. Our study might not have considered FSWs who do not want anyone to know they are FSWs due to several reasons, leading to a possible underestimation of the true PSE.

## Introduction

Increased risk for HIV transmission is highly associated with social marginalization, and those individuals who are socially marginalized may not identify themselves as such when accessing services. This makes it difficult to track them in HIV program registers and impedes efforts to plan and have informed resource allocations for high impact. Those individuals are considered to be key populations for HIV and include female sex workers (FSWs), men who have sex with men, transgender women, and people who inject drugs—all of whom are at increased risk for HIV infection compared with the population at large [1,2]. In 2021, key populations and their sexual partners accounted for 70% of HIV infections globally, with 51% in sub-Saharan Africa, and the risk of HIV acquisition among FSWs is 30 times higher than that in adult females globally [2-4].

Rwanda, an East African country, is surrounded by 4 neighboring countries: Tanzania, Uganda, Democratic Republic of the Congo, and Burundi. Rwanda is divided into 5 administrative regions—4 provinces and the City of Kigali—and 30 districts as another subnational unit level. Rwanda experiences a mixed HIV epidemic, generalized in the adult population, with an adult (age 15-49 years) HIV prevalence stabilized at around 2.6% and with aspects of a concentrated epidemic among specific key populations at higher risk of HIV infection, with 45% among FSWs [5]. In Rwanda, FSWs are considered among the key populations for HIV prevention and care in the HIV and AIDS National Strategic Plan (2018-2024) [6].

Since 2010, the Rwanda Biomedical Center (RBC) has conducted 3 rounds of street- and venue-based FSW population size estimations (PSEs) nationally. The first was implemented in 2010 [7], where 3 methods were used, namely, 2-source capture-recapture (CRC), enumeration, and the multiplier method [8]. Using CRC and enumeration methods yielded the FSW PSE slightly more than 3000 [7]. Two years later, in 2012, through a household survey, RBC estimated the size of FSWs to range from 25,000 to 45,000 in Rwanda [9]. Lastly, in 2018, RBC conducted a national exercise of FSW size estimation by using the 3-source CRC (3S-CRC) method, where findings showed FSW PSE to range from 8328 to 22,806 with a median of 13,716 [10].

The recent 5th Rwanda Population and Housing Census in 2022 reported an average annual growth rate of 2.3%, where the current resident population increased beyond 13 million from 10.5 million in 2012 [11]. This rapid increase in the general population reflects the need for regular update of key population size estimates to inform HIV programming and planning in Rwanda. As affirmed by the Joint United Nations Program on HIV/AIDS or World Health Organization in the publication guidelines for second generation HIV surveillance [12], HIV surveillance among key populations is a priority in all epidemic settings. Identifying the key population groups, their locations, and their sizes helps in understanding and prioritizing the current needs for HIV prevention, diagnosis, treatment, and care services. It also helps in projecting the future needs for those services. Rwanda’s HIV and AIDS National Strategic Plan (2018-2024) has adopted routine surveillance of key populations, including regularly conducting PSEs of FSWs every 2-3 years [13]. This study aims at providing for the fourth time the population size estimate of street- and venue-based FSWs and sexually exploited minors aged 15 years and older operating in Rwanda, where the 3S-CRC method was used for the second time.

## Methods

### Study Population

The study population consists of biologically born females (girls or women), aged 15 years and older, who self-reported having any type of sex with men in exchange for goods, money, or services in the last 3 months and practicing sex work at street- and venue-based hotspots. Those fulfilling the above criteria and who were younger than 18 years are herein referred to as sexually exploited minors.

### Study Design and Setting

This was a cross-sectional national FSW and sexually exploited minor PSE by using the 3S-CRC method [14]. The method involved visiting hotspots, where FSWs are known to congregate, on 3 separate occasions and sampling FSWs who were found at the hotspots on each occasion, calculating the degree to which FSW samples overlapped across 3 consecutive occasions. In this framework, an encountered FSW at the visited hotspot was referred to as captured, and each encounter occasion was referred to as a capture round in the CRC method context. A resampled FSW at a subsequent capture round was referred to as recaptured, and the intuition was that the degree to which FSW samples overlap across the 3 consecutive capture rounds was inversely proportional to the population size.

The objects used to tag FSWs who were presented at the hotspots were small, inexpensive, and branded with specific messages so that they would have a memorable design, and these would only be available from the study staff who distributed them. During the first capture, a small bag branded with the “imigongo” traditional art form was offered; for the second capture, a purse branded with a flower and the key message “Rinda ubuzima” (protect your life) was offered; and during the third capture, a hair comb branded with a tree picture as a key message was offered.

### Sampling and Sample Size

#### Sampling Design

A stratified multistage sampling design was used, with administrative provinces considered as strata and FSW hotspots as primary sampling units. Information from FSW’s hotspot mapping exercise was used as the sampling frame for this FSW PSE in 2022. Prior to this survey, the RBC conducted an FSW hotspot mapping exercise across the country from March to May 2022 to collect some key information that would inform future studies involving FSWs. Hotspot mapping consisted of teams going to the field to identify active venues and streets where FSWs congregate to find sexual clients. The FSW hotspot mapping exercise was facilitated by key informants identified by implementing partners who provide health services to FSWs to guide mapping teams. The mapping exercise identified 668 hotspots (street- and venue-based) countrywide and collected some beneficial data, including hotspot name, hotspot size, pick days, pick hours, and corresponding geocoordinates, to guide the sampling process.

The principal sampling processes were as follows: from the national list of FSW hotspots resulting from the hotspot mapping exercise, FSW hotspots were stratified by administrative provinces and the City of Kigali, and then a specific number of hotspots was selected using probability proportional to the number of FSW hotspots within each of the 4 provinces and the City of Kigali. Hotspot sampling was performed using probability proportional to size for generating samples. In probability proportional to size sampling, the probability that a hotspot was sampled was proportional to the estimated size of FSWs observed at that hotspot during the hotspot mapping exercise. In practice, this means that hotspots with many FSWs are more likely to be sampled than hotspots with fewer FSWs.

To enhance the geographical representativeness of the sample, hotspots were listed by the corresponding administrative provinces, and provinces were considered strata. To execute hotspot sampling, we listed all the hotspots in the order of the number of FSWs observed during the mapping exercise within a strata (to reflect the relative sizes of the FSW populations). Then, we calculated the cumulative number of FSWs for each hotspot listed, determined the sampling interval, picked a random starting point, and finally selected a hotspot based off the random starting point, sampling interval, and cumulative FSW population size. This process was repeated in each capture round to minimize list dependency between capture occasions, and this resulted in the selection of 62 hotspots countrywide in each capture round.

#### Sample Size Calculation

The expected sample size for each capture round and statistical power were calculated using MS-CRC Power Analysis of the shinyrecap application [15]. Using the previously estimated size of FSWs in Rwanda of 23,495 [10], we set the application to simulate 500 CRC studies and report the amount of variability in the estimates based on the posited population and the sample size in each capture event to 2000 at an α level of .05. We found that there is a 95% chance that the CRC study’s population size estimate will be within 7.6% of the true value, corresponding to 1780 absolute accuracy. Considering the 11% nonresponse rate from the previous study, 2000 was found to be an appropriate sample size for each capture round (Multimedia Appendix 1). The number of objects distributed to each hotspot was proportional to the total number of FSWs estimated at the hotspot according to the 2022 mapping data.

To select the number of FSWs to be offered unique objects within a selected FSW hotspot, we used a systematic sampling approach for the distribution of unique objects. The unique object distribution process started with the FSW key informant conducting visual head counts of FSWs present at the hotspot and then estimating the distribution interval by dividing the head counts by the assigned hotspot unique objects. If the result of the division was 1, every FSW present at the hotspot should have received the unique object; otherwise, a random start would be randomly selected within the distribution interval following the physical standing position of FSWs in the hotspot. Table 1 shows the provincial distribution of the sampled 62 hotspots and the 2000 unique objects assigned in each capture round.

**Table 1:** Provincial level sample size replicated at each of the 3 capture rounds in Rwanda in 2022.

Province	Information from hotspot mapping (n)		3-source capture-recapture sampling (n)		
	Hotspots per province (n=697)	Estimated total FSWs^a^ at hotspots during mapping exercise (n=22,471)	FSWs to be sampled (n=2000)	Hotspots to be selected and visited (n=62)	Average number of FSWs to be sampled per hotspot
City of Kigali	100	3883	346	9	39
East	237	5825	518	21	25
North	74	3095	275	7	42
South	61	2858	255	5	47
West	225	6810	606	20	30

^a^FSW: female sex worker.

### Data Collection

Data collection was conducted in sampled hotspots and lasted for a 3-week period from August 1 to August 21, 2022. All data collectors were trained about questionnaire administration using tablets as well as good clinical practice [16]. The 3 captures followed each other consecutively, each lasting for 1 week. Two data collectors from the 32 trained data collectors were randomly assigned to 28 districts (2 districts did not meet the minimum requirement to be included in the survey) of Rwanda in a team of 2 and shuffled on every capture round. Once they arrived in the selected hotspots, the data collection team was assigned a local FSW guide (key informant) who helped in object distribution among FSWs who were congregated in the selected hotspots. The hotspot visiting time depended on the selected pick days and hours for each venue or street, and the objects were distributed systematically by determining the interval according to the number of FSWs present at the hotspots and the number of assigned unique objects.

The unique objects were distributed by FSW key informants under the supervision of data collectors, and the latter only recorded responses of whether the approached FSW accepted or refused the unique object. Those who accepted the unique object were marked as successful capture. For the successful captures, FSWs were asked if they had received a previously distributed unique object, and this information was also recorded on the tablet. If a successfully captured FSW claims to have received the previously distributed unique object, then she was asked to present it to the key informant in case she had it with her. Otherwise, a laminated card with different unique objects, including the correct distributed unique objects, was shown to her to point out the object she claimed to have received, and the data collector recorded if she successfully pointed out the correct unique object. As different unique objects were distributed in each capture round, depending on the type of object presented physically or pointed out on the laminated card, the data collector would mark the corresponding capture round from which that object was distributed on the tablet. Furthermore, a visual estimation of the age group for the approached FSWs was recorded. Data were collected at individual level encounters, that is, for each approached FSW, a record would be opened, filled, and saved in the tablet before moving forward to the next FSW.

In summary, 4 major assumptions must be met for the CRC to give reliable population estimates; these were considered during data collection. These assumptions include that individual captures should be independent, the population should be closed during the data collection period, each target population member’s capture history should be correct, and the chance of getting captured should be homogeneous [17]. To minimize dependencies between captures, we repeated the sampling process of FSW hotspots in each capture round. To reduce recall bias and to ensure that the closed population assumption was met for all 3 captures, we maintained a 1-week period between consecutive capture rounds. Within sampled FSW hotspots, FSWs were sampled systematically to receive the distributed unique objects to ensure that the probability of being sampled was homogeneous. For details, please refer to the sampling and sample size section above. Finally, data collection was monitored in real time to ensure data quality and to ensure that each individual capture history was correct.

### Data Management

Data were collected electronically using a tablet questionnaire programmed in Open Data Kit and transferred to the central server, with the process monitored by a qualified data manager. Electronic data files, computers, and other storage devices that contained data were password-protected, and electronic survey data files had encryption protection. Data files were transferred to the central encrypted server immediately after individual encounters. If the internet connection was not strong enough to upload data to the server, the records would be backed up on the tablet and sent later once the team reached the area with strong internet connectivity.

Data collectors were trained on the process of data collection and the use of tablets to ensure data quality. All data were anonymized, and no personal identifying information was collected. Participant-level data were line-listed and uniquely identified by a tablet-generated unique study ID. Data extracted from the central server were transferred into Microsoft Excel, maintained on a password-protected computer, and backed up on an external hard drive to ensure the security of the data, and kept in a locked, secure location. On a daily basis, the data manager who had access to the secure data would download the received data in Excel and conduct a quality check to ensure that high-quality data standards were met, including data logical flow and skip patterns. Whenever an error was identified, the data manager would immediately reach out to the concerned data collector for clarifications and rectify the error.

### Statistical Analysis

Data cleaning was conducted using STATA 17 and RStudio (shinyrecap) for 3S-CRC for data analysis [18]. In preparation for the analysis, participant-level data were exported into RStudio (R package: shinyrecap) for Windows, and cleaning was performed based on preset exclusion criteria (self-reporting to be an FSW and accepting the offered unique object) and data logical flow following skip patterns. The data set was subset by province to have provincial-level FSW population size estimates. Aggregated data sets detailing counts of each CRC combination were produced for each subset. Table 2 shows how data were aggregated by overall and provincial 2^k^ – 1 contingency tables for analysis preparation, where k stands for the number of capture occasions and n_i_ represents aggregated counts, where i stands for a specific capture occasion.

**Table 2:** Three-source capture-recapture aggregated data set of Rwanda in 2022.

Capture 1	Capture 2	Capture 3	Total
1	0	0	n_1_
0	1	0	n_2_
0	0	1	n_3_
1	1	0	n_1&2_
1	0	1	n_1&3_
0	1	1	n_2&3_
1	1	1	n_1&2&3_

Frequentist log-linear models [19], Bayesian nonparametric latent class [20], and Bayesian Model Averaging [21], which are flexible and able to accommodate various forms of heterogeneity in capture probabilities, were used to produce the final PSE with credibility sets from aggregated data sets. The median population size with 95% credibility sets and confidence intervals for 3S-CRC data were produced overall and by province. The selection of the best model to report among the 3 mentioned ones was based on whether the data presented list dependency (capture events independently drawn from the population) of captures or capture heterogeneity (individuals in the population have the same probability of being captured).

### Ethics Approval

The survey received ethics approval from the Rwanda National Ethics Committee (IRB00001497). It was also reviewed in accordance with the US Centers for Disease Control and Prevention human research protection procedures and was determined to be not research. A waiver for consent was obtained, and no compensation was offered, as data collectors did not interact with the participants. The survey protected the anonymity of participants to avoid any stigmatization, and no personally identifiable information was collected. A referral form was available for sexually exploited minors, which included referrals to health and legal services.

## Results

Of the 1778 FSWs approached during capture 1, 1768 (99.4%) were newly captured (ie, they were not captured elsewhere within the same week). Among those newly captured, unique object acceptance was high at 99.9% (1766/1778). For 1870 FSWs approached during capture 2, 1851 (98.9%) were newly captured within the second week of capture. Among those newly captured in capture 2, unique object acceptance was high at 99.8% (1848/1851). During capture 3, 1910 FSWs were approached, and 1867 (97.7%) were newly captured. The main reasons for unique object refusal documented were not being willing to receive the object and being willing to receive money instead of a unique object. Table 3 presents the results by capture round.

The majority of the FSWs sampled were presumed to be 25 years old, while only few sexually exploited minors aged 15-17 years were captured across all 3 capture rounds. Table 4 describes the sampled FSWs in each capture round by age and province.

**Table 3:** Results of the 3-source capture-recapture by capture round during female sex worker population size estimation in Rwanda in 2022.

			Capture 1		Capture 2		Capture 3
Approached female sex workers (n)			1778		1870		1910
**Already in current capture, n (%)**							
	Yes	10 (0.6)		19 (1.1)		43 (2.3)	
	No	1768 (99.4)		1851 (98.9)		1867 (97.7)	
**Unique object acceptance, n (%)**							
	Accepted	1766 (99.9)		1848 (99.8)		1865 (99.9)	
	Refused	2 (0.1)		3 (0.2)		2 (0.1)	
**Reason for refusal (n)**							
	Does not want or refused unique object	1		1		2	
	Wanted money and not objects	1		2		0	

**Table 4:** Sampled female sex workers by capture round, age group, and province as per the female sex worker population size estimation in Rwanda in 2022.

Capture, age group (years)		City of Kigali (n)	Eastern province (n)	Northern province (n)	Southern province (n)	Western province (n)
**Capture 1 (n=1766)**						
	15-17 (n=28)	9	2	2	2	13
	18-24 (n=628)	122	109	162	106	129
	25+ (n=1110)	127	142	222	247	372
**Capture 2 (n=1848)**						
	15-17 (n=11)	5	5	5	0	0
	18-24 (n=911)	116	130	206	126	329
	25+ (n=926)	239	164	162	124	237
**Capture 3 (n=1865)**						
	15-17 (n=35)	0	5	3	3	24
	18-24 (n=851)	184	155	85	92	335
	25+ (n=979)	131	152	276	142	278

**Figure 1:**
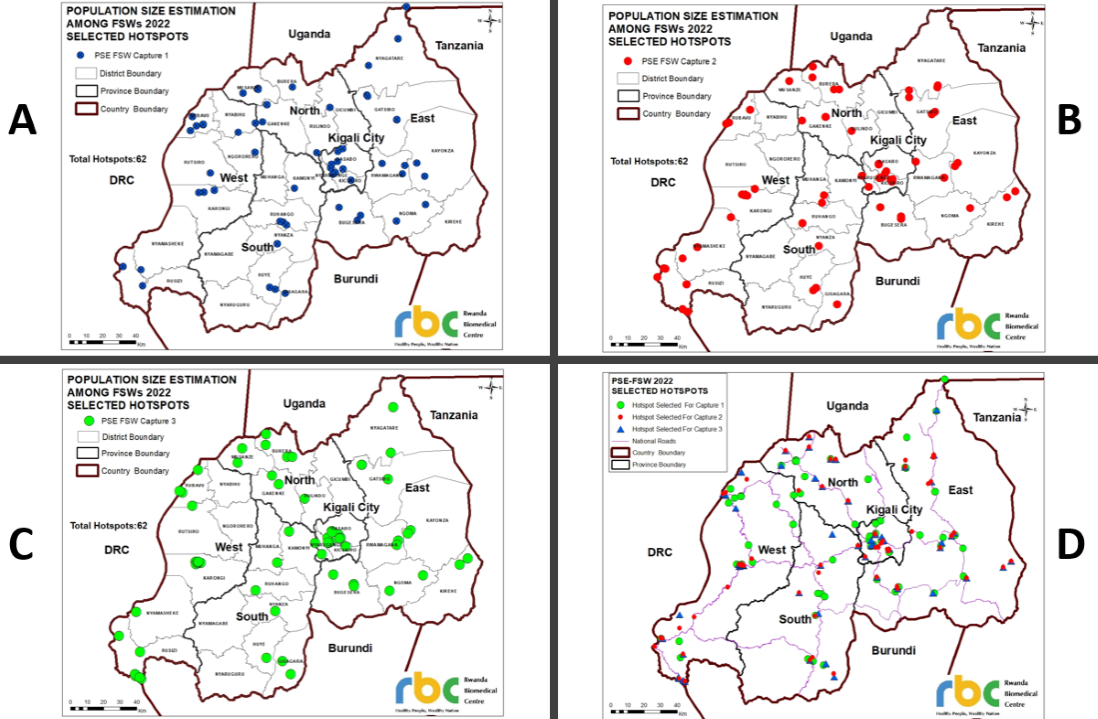
Maps of individual captures highlighting the venue street hotspots visited in Rwanda in 2022. Map A: capture 1. Map B: capture 2. Map C: capture 3. Map D: All 3 captures combined. DRC: Democratic Republic of the Congo; FSW: female sex worker; PSE: population size estimation.

A total of 1766 unique objects were distributed countrywide during capture 1, 1848 objects during capture 2, and 1865 objects during capture 3. In a 3-week survey implementation exercise, 62 hotspots were visited countrywide in each capture round; however, bigger hotspots were resampled in the subsequent capture rounds. Two hotspots were resampled between capture 1 and capture 2; 8 hotspots were resampled between capture 2 and capture 3; 6 hotspots were resampled between capture 1 and capture 3; and 2 hotspots were resampled in all 3 capture rounds. Figure 1 shows the maps of the individual captures, highlighting the venue and street hotspots visited. The aggregated and cleaned final 3S-CRC data set was imported into shinyrecap for analysis.

**Figure 2:**
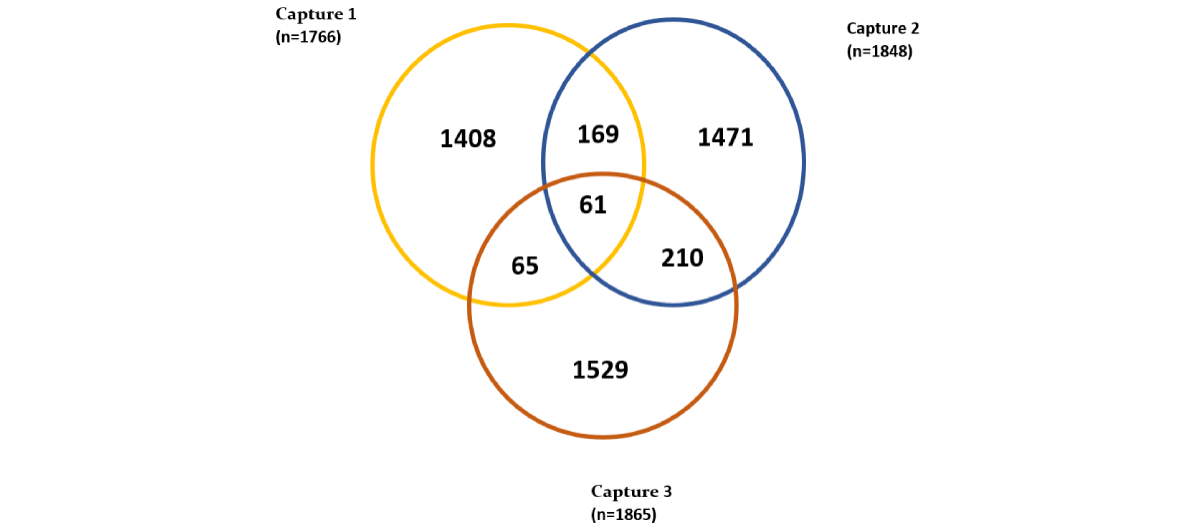
Venn diagram presenting the national aggregated data of capture history results for single, double, and triple captures in Rwanda in 2022.

For all 3 capture rounds, 1766 FSWs, 1848 FSWs, and 1865 FSWs were sampled, of which 1408 FSWs, 1471 FSWs, and 1529 FSWs were observed strictly during capture 1, capture 2, and capture 3, respectively. There were 169 exclusive overlaps between capture 1 and capture 2, 210 exclusive overlaps between capture 2 and capture 3, and 65 recaptures between capture 1 and capture 3. Finally, 61 FSWs were recaptured in all 3 capture rounds. Figure 2 presents the Venn diagram illustrating the aggregated data of the capture history results for single, double, and triple captures to construct a structured 3S-CRC data set.

Out of the 231 FSWs recaptured between capture 1 and capture 2, 96 physically presented the unique objects received during capture 1, while out of 135 who did not have the unique objects with them, 134 were able to correctly describe and identify the received unique object on a laminated card, bringing the total number of recaptures to 230. Of the 127 FSWs recaptured between capture 1 and capture 3, 53 brought the unique objects with them, while of the 74 who did not have the unique objects with them, 73 were able to correctly describe and identify the received unique object on a laminated card. Of the 272 FSWs recaptured between capture 2 and capture 3, 111 had the received unique objects with them, while 160 who did not have the unique objects with them were able to correctly describe and identify the received unique object on a laminated card. Table 5 highlights the 2 methods used to record the recapture histories.

**Table 5:** The recapture identification cascade in the female sex worker population size estimation in Rwanda in 2022.

		Recapture round (n)	
		Capture 2	Capture 3
**Capture 1 (C1)**			
	Total recaptured from C1	231	126
	Showed C1 object	96	53
	Did not have unique objects with them	135	74
	Correctly identified C1 object	134	73
**Capture 2 (C2)**			
	Total recaptured from C2	N/A^a^	271
	Showed C2 object	N/A	111
	Did not have unique objects with them	N/A	161
	Correctly identified C2 object	N/A	160

^a^N/A: not applicable.

Out of the 135 FSWs who claimed to have been offered capture 1 unique object during capture 2, only 1 was unable to describe and correctly identify the object received on the laminated card. Out of the 74 FSWs in capture 3 who claimed to have been offered a unique object but who did not have the objects with them, 73 were able to describe and correctly identify the object received on the laminated card. Only 1 FSW out of 272 FSWs who claimed to have been offered capture 2 unique object during capture 3 was unable to describe and correctly identify the object received on the laminated card. The FSW population size presented in Table 6 is based on 3 models: log-linear, Bayesian Model Averaging (using noninformative prior), and Bayesian nonparametric latent class models.

**Table 6:** National population size estimates of female sex workers aged ≥15 years by using the 3-source capture-recapture method during the population size estimation in Rwanda in 2022.

Model type	Proportion (%) of women (95% CI)^a^	Median population size estimation (95% credible set)
Log-linear (Mth Poisson2)^b^	1 (0.8-1.2)	34,370 (28,164-42,246)
Bayesian Model Averaging (noninformative prior)	1.1 (0.9-1.3)	37,647 (31,873-43,354)
Bayesian Latent Class	1 (0.4-1.6)	35,954 (14,736-55,215)

^a^Denominators are the national total number of adult females aged 15 years and older in the 5th Rwanda Population and Housing Census in 2022.

^b^Poisson model, which assumes that captures may have different probabilities and that individuals may be heterogeneous.

Based on the outputs and model diagnostics (Multimedia Appendices 1-4), the data were found to contain list dependence; therefore, Bayesian Model Averaging with noninformative prior was chosen, which best dealt with list dependence, as it automatically detected potential dependencies in the data. After fitting the model, the population size of street- and venue-based FSWs in Rwanda was estimated to be within a credible set ranging from 31,873 to 43,354 with a median of 37,647, corresponding to 1.1% (95% CI 0.9%-1.3%) of the general population of adult females aged 15-49 years in Rwanda (Table 7). Relative to adult females in the general population, the western and northern provinces ranked first and second with a higher concentration of FSWs, respectively. The City of Kigali and eastern province ranked third and fourth, respectively. The southern province was identified as having a lower concentration of FSWs.

**Table 7:** Female sex worker provincial population size estimates produced using Bayesian Model Averaging with noninformative prior population size estimation in Rwanda in 2022.

Province	Proportion (%) of women 15-49 years who were female sex workers (95% CI)^a^	Median population size estimation (95% credible set)
City of Kigali	0.8 (0.5-1)	3974 (2815-5197)
Eastern province	0.6 (0.3-1)	5022 (2535-8601)
Northern province	1.1 (0.7-1.6)	5993 (3710-8876)
Southern province	0.5 (0.2-0.9)	3884 (1548-6727)
Western province	1.2 (0.9-1.6)	8983 (6536-11,791)

^a^Denominators are the provincial total number of adult females aged 15 years and older in the 5th Rwanda Population and Housing Census in 2022.

## Discussion

This study provides both national and provincial-level estimates of the population size of street- and venue-based FSWs and sexually exploited minors aged 15 years and older in Rwanda. In our study, the population size of street- and venue-based FSWs and sexually exploited minors was estimated to be within a credible set ranging from 31,873 to 43,354, with a median of 37,647, corresponding to 1.1% (95% CI 0.9%-1.3%) of the adult females aged 15 years and older in the general population. Our results indicate a significant difference in the FSW population size as compared to the 2018 population size of FSWs aged 15 years and older, which was estimated to range from 8328 to 22,806 credible sets with a median of 13,716 [10]. This difference may be attributed to several factors, including but not limited to differences in the estimation models used and the geographical coverage.

Furthermore, our study provides a provincial-level population size estimate of street- and venue-based FSWs and sexually exploited minors aged 15 years and older for the very first time. The largest population size estimate was obtained in the western province, followed by the northern and eastern provinces. The City of Kigali and southern province were found to have relatively lower estimates of the FSW population as compared to other provinces. Differences in the estimates distribution across the country may reflect long-term internal movement patterns among FSWs, from rural to more urbanizing areas as well as from smaller to larger urbanized contexts, as indicated by the Rwanda Population and Household Census 2022 [22].

The findings from the 2022 FSW PSE might not have considered high-profile FSWs and those FSWs using web-based and social media platforms to reach their clients, leading to a slight possible underestimation of the true population size. Furthermore, we acknowledge possible methodological limitations that might influence the final FSW PSE in this study. Compared to the program coverage data of the Rwanda Health Management Information System, the key strength of our study is that it is powered to provide national and provincial-level PSE for FSWs in Rwanda for the very first time.

So far, 3 rounds of FSW PSE have been conducted in Rwanda since 2010 [7,9,10]. The 2010 FSW size estimation using CRC and multiplier methods estimated the national population size of FSWs to range from 2998 to 3412 with a median of 3205. In 2012, the population size of FSWs was estimated to range from 23,000 to 39,000. Later, after 6 years, in 2018, the national population size of FSWs was estimated to range from 8328 to 22,806, with a median of 13,716. These differences in the population size of FSWs might be attributed to different reasons, including but not limited to methodological or geographical coverage differences. Compared with the previous 3 rounds of FSW PSE exercises, we observed a difference in the FSW population size in our study, which also might be attributed to the reasons stated above.

There are several methods to estimate the population size of population groups without sampling frames [23,24]. Each method presents its own unique strengths and weaknesses. Our study uses the 3S-CRC method to produce FSWs and sexually exploited minors’ PSEs nationally and at the subnational level for 5 administrative provinces. Two captures are used in the classic CRC method; however, additional captures can be added to increase the number of data points from which estimates are generated, resulting in increased ability to account for the potential interaction; so, the assumption of independence between captures may be relaxed [20]. Due to its mathematically grounded and logical results, 3S-CRC is increasingly utilized in epidemiology to estimate the size of the key populations targeted by health intervention programs for certain health disorders [25-27]. The 3S-CRC approach has been utilized in numerous studies to estimate the size of specific population groups—including FSWs, men who have sex with men, and people who inject drugs—without sampling frames [28-32].

As affirmed by the Joint United Nations Program on HIV/AIDS or World Health Organization in the publication guidelines for the second generation Know Your HIV epidemic [12], HIV surveillance among key populations is a priority in all epidemic settings. Identifying the key population groups, their locations, and their sizes helps in understanding and prioritizing the current needs for HIV prevention, diagnosis, treatment, and care services. It also helps in projecting the future needs for those services.

Despite the limitations mentioned above, policy makers and planners should be able to monitor HIV pandemic control nationwide, specifically among FSWs, by using the findings from the 2022 PSE of FSWs. They should also be able to plan for other health services such as sexually transmitted infection prevention and treatment. Even though these estimates can be applied at the national and provincial levels, more work on small area estimation is needed to match PSE results with the targeted HIV treatment and prevention efforts at lower subnational levels. Further, we admit that some FSW groups can still be difficult to estimate, including but not limited to those reaching their clients on web-based platforms; this could be a topic for future research.

## Appendix

Multimedia Appendix 1 Multiple capture-recapture power analysis.

Multimedia Appendix 2 Log-linear analysis for producing the final population size estimate with credibility sets from aggregated data sets in Rwanda in 2022.

Multimedia Appendix 3 Bayesian Latent Class for producing the final population size estimate with credibility sets from aggregated data sets in Rwanda in 2022.

Multimedia Appendix 4 Bayesian Model Averaging for the final population size estimate in Rwanda in 2022.

## Abbreviations

3S-CRC: 3-source capture-recapture
CRC: capture-recapture
FSW: female sex worker
PSE: population size estimation
RBC: Rwanda Biomedical Center

## Data Availability

The data that inform the findings in this manuscript are client-level data fully owned by the Rwanda Ministry of Health and the Rwanda Biomedical Center. The data cannot be shared publicly to protect client privacy and confidentiality, considering that female sex workers is a sensitive key population group. However, researchers who meet set criteria can access the data upon request and approval by the Rwanda Biomedical Center (data access requests should be sent to info@rbc.gov.rw).
