# Supplementary figures and images for: Estimation of the Population Size of Street- and Venue-Based Female Sex Workers and Sexually Exploited Minors in Rwanda in 2022: 3-Source Capture-Recapture
Source: JMIR Public Health Surveill. 2024 Mar 15;10:e50743. doi: 10.2196/50743 (PMC10980986; doi:10.2196/50743)

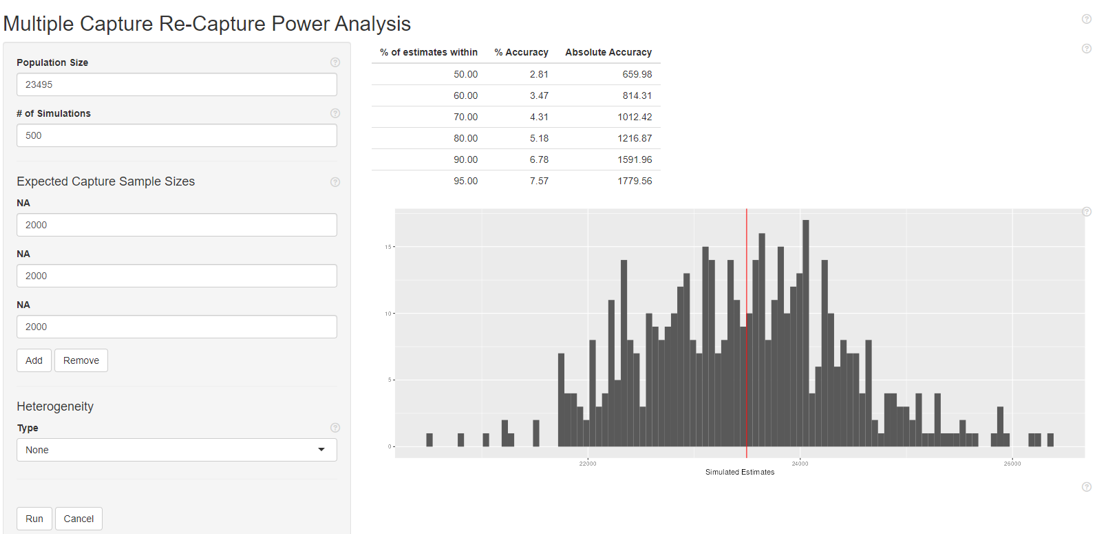

Supplement: Multimedia Appendix 1 [file publichealth_v10i1e50743_app1.png]

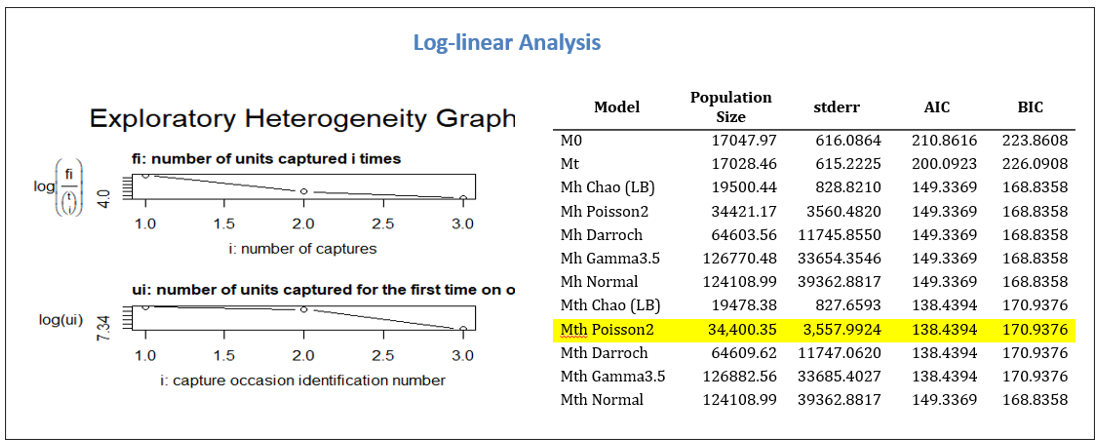

Supplement: Multimedia Appendix 2 [file publichealth_v10i1e50743_app2.png]

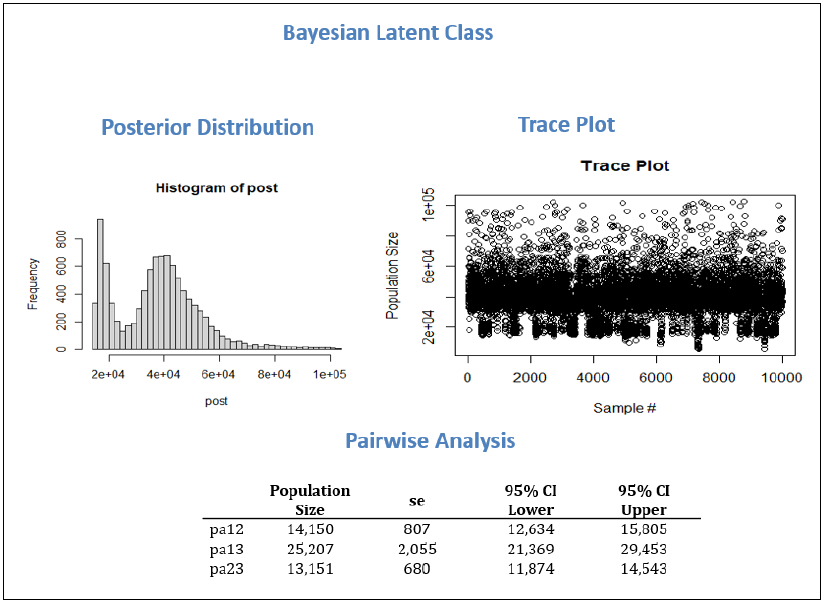

Supplement: Multimedia Appendix 3 [file publichealth_v10i1e50743_app3.png]

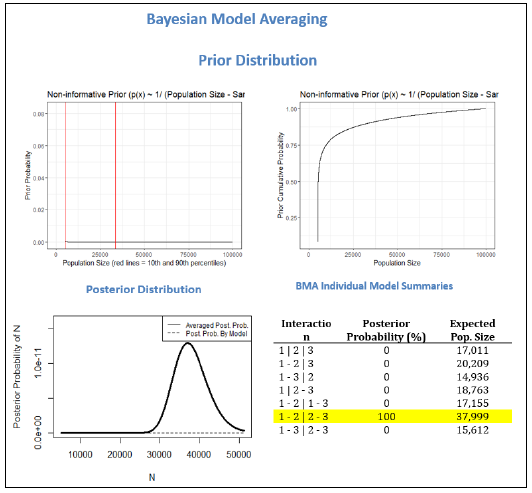

Supplement: Multimedia Appendix 4 [file publichealth_v10i1e50743_app4.png]
